# Indoleamine-2,3-dioxygenase activity in experimental human endotoxemia

**DOI:** 10.1186/2040-7378-4-24

**Published:** 2012-12-05

**Authors:** Jan-Sören Padberg, Matijs Van Meurs, Jan T Kielstein, Jens Martens-Lobenhoffer, Stefanie M Bode-Böger, Jan G Zijlstra, Csaba P Kovesdy, Philipp Kümpers

**Affiliations:** 1 Department of Medicine D, Division of General Internal Medicine, Nephrology, and Rheumatology, University Hospital Münster, Albert-Schweitzer-Campus A1, Münster 48149, Germany; 2 Department of Critical Care, University Medical Center Groningen, Hanzeplein 1, University of Groningen, Groningen, GZ, 9713, The Netherlands; 3 Department of Pathology and Medical Biology, University Medical Center Groningen, Hanzeplein 1, University of Groningen, Groningen, GZ, 9713, The Netherlands; 4 Department of Nephrology & Hypertension, Hannover Medical School, Carl-Neuberg-Strasse 1, Hannover, 30625, Germany; 5 Institute for Clinical Pharmacology, Otto-von-Guericke University Magdeburg, Leipziger Straße 44, Magdeburg, 39120, Germany; 6 Divisions of Nephrology, University of Virginia, Charlottesville, Virginia & Salem VA Medical Center 1970 Roanoke Blvd, Salem, VA, 24153, USA

## Abstract

**Background:**

Excessive tryptophan metabolism to kynurenine by the rate-limiting enzyme endothelial indoleamine 2,3-dioxygenase 1 (IDO) controls arterial vessel relaxation and causes hypotension in murine endotoxemia. However, its relevance in human endotoxemia has not been investigated so far. We thus aimed to study changes in blood pressure in parallel with tryptophan and kynurenine levels during experimental endotoxemia in humans.

**Findings:**

Six healthy male volunteers were given E. coli lipopolysaccharide (LPS; 4 ng/kg) as a 1-min intravenous infusion. They had levels of soluble E-Selectin and soluble vascular cell adhesion molecule-1 as well as IDO activity assessed as the kynurenine-to-tryptophan plasma ratio by liquid chromatography-tandem mass spectrometry at various time points during a 24 h time course. During endotoxemia, IDO activity significantly increased, reaching peak levels at 8 h after LPS infusion (44.0 ± 15.2 vs. 29.4 ± 6.8 at baseline, P<0.0001). IDO activity correlated inversely with the development of hypotension as shown by random effects linear regression models. Finally, IDO activity exhibited a kinetic profile similar to that of soluble endothelial-specific adhesion molecules.

**Conclusions:**

LPS is a triggering factor for the induction of IDO in men. Our findings strongly support the concept that the induction of IDO in the vascular endothelium contributes to hypotension in human sepsis.

## Introduction

Loss of vascular tone is an important factor in the development of severe sepsis, septic shock and multiple organ failure. Recent work by Wang and colleagues provided experimental evidence that kynurenine, a metabolite of the amino acid L-tryptophan, is a mediator of vasodilation and subsequent hypotension during murine endotoxemia [1]. In different mouse models of sepsis and additional in vitro experiments, they found that kynurenine acts as a novel endothelium-derived relaxing factor on resistance vessels by stimulating the activity of soluble guanylyl cyclase and adenylyl cyclase in smooth muscle cells. In addition the authors could show that injecting mice with bacterial lipopolysaccharide (LPS) specifically induces the rate-limiting enzyme for kynurenine production, namely indoleamine 2,3-dioxygenase 1 (IDO), in vascular endothelial cells [1]. Consistent with these findings, pharmacological blockade or genetic deletion of IDO increases survival in murine endotoxemia [2]. However, animal studies are potentially confounded by major inter-species differences in the sensitivity and immune response to various types of inflammatory stimuli [3]. The aim of this study was thus to correlate temporal changes in IDO activity (plasma kynurenine/L-tryptophan ratio) with the onset of hypotension in humans. Therefore, we re-measured plasma kynurenine and L-tryptophan, and calculated the IDO activity in serial samples from a well-defined human endotoxemia model [4,5].

## Materials and methods

### Endotoxemia model

Six healthy male subjects, aged 32 ± 4 years, were admitted to the research unit of our intensive care department. The local medical ethics committee (Medisch Ethische Toetsingsingscommissie, METc, of the University Medical Center Groningen, NL) approved the study and written informed consent was obtained from all subjects before enrolment. Subjects were admitted 15 h prior to endotoxin infusion. A radial artery catheter was placed for blood sampling and arterial blood pressure measurement. During the study, systolic blood pressure (SBP), diastolic blood pressure (DBP), mean arterial pressure (MAP) and pulse rate were measured continuously. At time point zero (0 h) the volunteers received a 1-min infusion of endotoxin (Escherichia coli LPS, batch EC-6, US Pharmacopeia, Twinbrook Parkway, Rockville, MD, USA) at a dose of 4 ng/kg body weight (10,000 endotoxin units/μg) via an indwelling venous catheter. Blood samples for marker analysis were obtained pre-dose and at several time points up to 24 h after endotoxin infusion. All samples were immediately placed on ice, centrifuged (1,500G, 15 min, 4°C) and stored at -70°C until analysis. Data from this study have been reported extensively elsewhere [4,5].

### Quantification of tryptophan, kynurenine, and soluble adhesion molecules

Levels of tryptophan and kynurenine were determined using a liquid chromatography-tandem mass spectrometry (LC-MS/MS) method utilizing deuterated internal standards for both analytes. The method was adapted from de Jong et al. [6]. In short, plasma samples were prepared for analysis by protein precipitation with acetonitril. After centrifugation, the clear supernatant was stripped from the acetonitrile portion by vacuum centrifugation and the aqueous residue was injected into the LC-MS/MS system. Chromatographic separation was achieved on a porous graphitic carbon column with the dimensions 50 × 2 mm by a short step gradient (0.1% trifluoroacetic acid in water: acetonitrile 90:10, in 1 min to 50:50, 0.25 ml/min flow rate). Calibration ranges were 10-200 μmol/l for tryptophan and 0.5-10 μmol/l for kynurenine, respectively. Relative standard deviations were less than 5% for both substances at all quality control levels. IDO activity was calculated (IDO_c_) as the kynurenine to tryptophan ratio x 1000. Soluble E-Selectin (sEsel) and soluble vascular cell adhesion molecule-1 (sVCAM-1) levels were measured using *Fluorokine*® *MultiAnalyte Profiling* kits and a *Luminex*® Bioanalyzer (R&D Systems, Oxon, U.K.) according to the manufacturers’ instructions and have been reported elsewhere [7].

### Statistical analysis

Continuous variables are expressed as means ± one standard deviation (SD). A repeated-measures one-way analysis of variance with Dunnett’s test for multiple comparisons (two-sided) was used to demonstrate statistical changes in hemodynamic and laboratory variables after endotoxin infusion. Two-sided p-values < 0.05 were considered statistically significant. The association between IDO_c_ activity and hemodynamic parameters measured repeatedly at various time points in the same person was examined in random effects linear regression models treating hemodynamic parameters as dependent variables and using maximum likelihood estimators. The association between adhesion molecules and IDO_c_ activity was assessed in a similar manner, treating IDO_c_ activity as dependent variable. To fulfil the assumptions needed for the analysis, logarithmic (ln) transformation of sEsel was performed. Results are shown as beta-coefficients that correspond to a one SD higher value of each of the independent variables in each regression.

## Results

**Figure 1:**
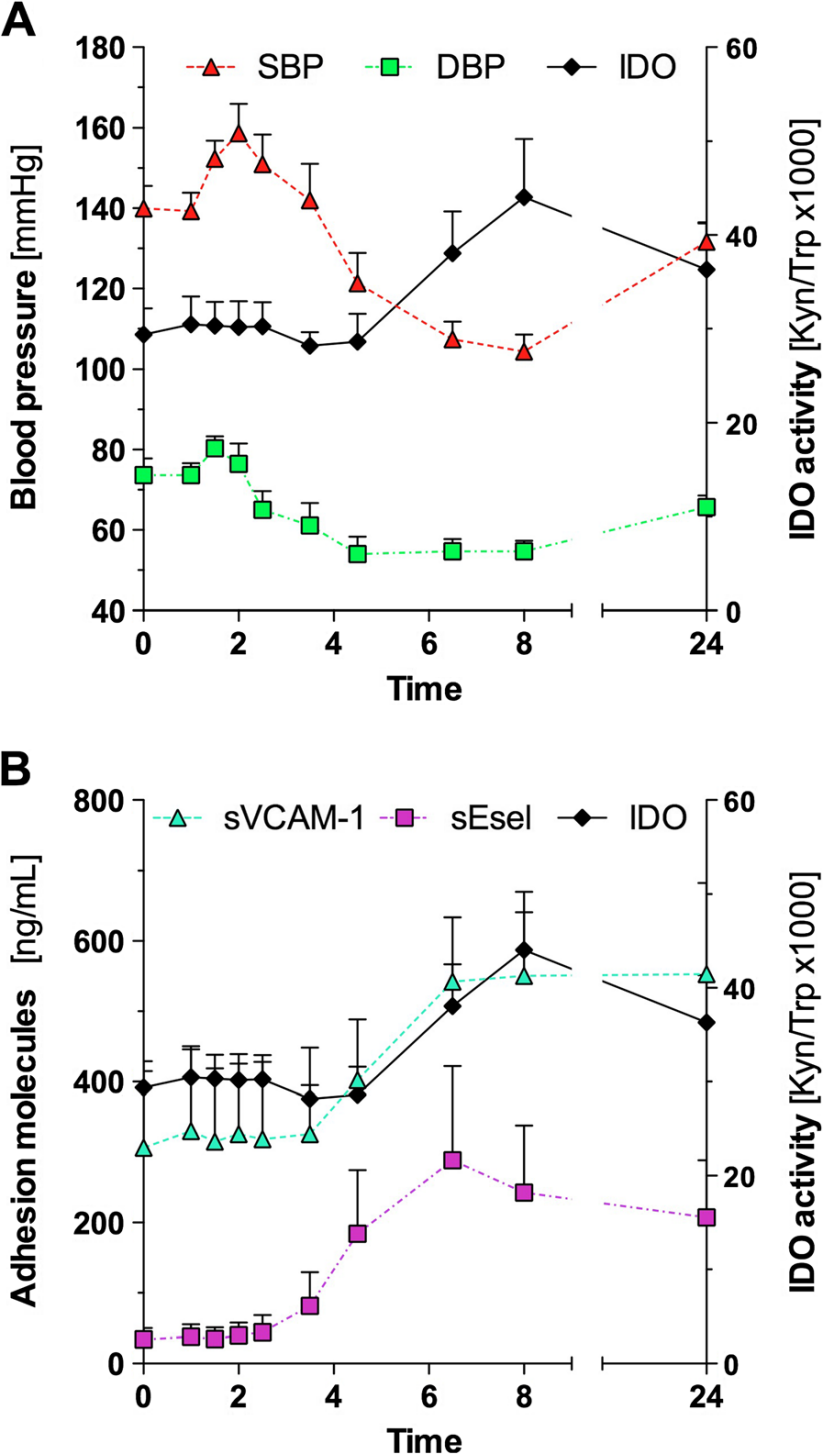
**Time course of blood pressure, endothelial inflammation, and indoleamine 2,3-dioxygenase 1 activity during human endotoxemia. **Activity of Indoleamine 2,3-dioxygenase 1 (IDO), calculated as the kynurenine to tryptophan ratio x 1000, compared with **A**) systolic blood pressure (SBP) and diastolic blood pressure (DBP) and **B**) plasma levels of endothelial soluble adhesion molecules E-selectin (sEsel) and vascular-cellular adhesion molecule-1 (sVCAM-1) after endotoxin infusion in six healthy volunteers.

LPS infusion elicited a significant laboratory and clinical inflammatory response with chills, headache, muscle pain, increased heart rate, and overt hypotension (Table 1). In brief, SBP was 140 ± 14 mmHg at 0 h, minimal at 8 h (104 ± 11 mmHg) and remained below baseline until 20 h (Figure 1A). Details on additional hemodynamics are given in Table 1. Two volunteers received 1000 mL of 0.9% saline i.v. due to a SBP below 70 mmHg. No vasopressors were needed. At 24 h, all volunteers were asymptomatic and all clinical parameters within the normal range. Consistent with maximal hypotension, IDO_c _activity significantly increased at 6.5 h and peaked at 8 h (44.0 ± 15.2 vs. 29.4 ± 6.8 at baseline, P<0.0001, Figure 1A). In random effects linear regression models, IDO_c_ activity was significantly associated with the degree of hypotension (SBP: β = -9.948 (95% confidence interval [CI] -16.361 to -3.535), p = 0.002; DBP: β = -3.907 (95% CI -7.448 to -0.336), p = 0.031; MAP: β = -5.725 (95% CI -9.922 to -1.529), p = 0.007).

**Table 1:** Time course after LPS infusion in healthy volunteers

			**Time course after LPS infusion**								
**Variables**	**Pre-Dose**	**1 h**	**1.5 h**	**2 h**	**2.5 h**	**3.5 h**	**4.5 h**	**6.5 h**	**8 h**	**24 h**	**P-value**
**HEMODYNAMICS**											
**SBP** (mmHg)	140 ± 14	139 ± 11	152 ± 11	158 ± 18*	151 ± 18	142 ± 22	121 ± 18*	107 ± 11*	104 ± 11*	131 ± 11	<0.0001
**DBP** (mmHg)	74 ± 10	74 ± 7	80 ± 7	77 ± 12	65 ± 12	61 ± 14*	54 ± 11*	55 ± 8*	55 ± 7*	66 ± 7	<0.0001
**MAP** (mmHg)	96 ± 11	96 ± 9	104 ±7	104 ± 13	94 ± 14	88 ± 16	77 ± 13*	72 ± 8*	71 ± 7*	88 ± 7	<0.0001
**Heart rate** (bpm)	61 ± 16	59 ± 11	78 ± 19	78 ± 18	92 ± 12	98 ± 8	101 ± 8	97 ± 11	96 ± 13	81 ± 16	<0.0001
**HR/MAP index**	0.64 ± 0.11	0.62 ± 0.11	0.75 ± 0.17	0.76 ± 0.22	1.01 ± 0.22*	1.15 ± 0.26*	1.36 ± 0.32*	1.36 ± 0.27*	1.4 ± 0.22*	0.92 ± 0.16*	<0.0001
**TRYPTOPHAN METABOLISM**											
**IDOc activity**	29.4 ± 6.8	30.5 ± 7.4	30.3 ± 6.2	30.2 ± 6.8	30.2 ± 6.4	28.2 ± 3.6	28.6 ± 7.2	38.1 ± 10.9*	44.0 ± 15.2*	36.3 ± 12.3*	<0.0001
**ADHESION MOLECULES**											
**sEsel** (ng/ml)	34 ± 16	38 ± 18	35 ± 16	40 ± 18	44 ± 24	82 ± 48	184 ± 90*	289 ± 133*	243 ± 95*	208 ± 81*	<0.0001
**sVCAM-1** (ng/ml)	307 ± 108	330 ± 120	315 ± 103	326 ± 100	319 ± 109	326 ± 122	403 ± 86*	542 ± 92*	550 ± 91*	553 ± 130*	<0.0001

A repeated-measures ANOVA with Dunnett’s test for multiple comparison (two sided) was used to demonstrate statistical changes in clinical and laboratory variables during the time course after endotoxin infusion (n=6). Two-sided p-values <0.05 were considered statistically significant. SBP – systolic blood pressure; DBP – diastolic blood pressure; MAP mean arterial blood pressure; HR/MAP index - heart rate/mean arterial pressure index; sEsel – soluble E-selectin; sVCAM-1 – soluble vascular cell adhesion molecule-1; IDO - Indoleamine 2,3-dioxygenase 1; *statistically significant vs. Pre-Dose.

Wang et al. [1] reported that IDO is specifically expressed induced in the inflamed endothelium of septic mice. Consistent with this notion, the increase in sVCAM-1 (P<0.0001) and soluble E-Selectin (P<0.0001), surrogates for endothelial inflammation, exhibited a kinetic profile similar to that of IDO_c_ in our human endotoxemia model (Figure 1B). IDO_c_ activity was tightly associated with plasma levels of sVCAM-1 (β = 5.07 (95% CI 3.25 to 6.9); p < 0.0001) and sEsel (β = 3.62 (95% CI 1.86 to 5.38); p < 0.0001) using random effects linear regression.

## Discussion

Here we show that, as in mice [1], LPS is a triggering factor for the induction of IDO in humans. Consistent with experimental findings by Wang and colleagues [1], we could show that IDO_c_ was associated with the extent of endothelial inflammation and correlated inversely with the development of hypotension. In support of our findings, Changsirivathanathamrong et al. [8] convincingly demonstrated in a clinical study, that IDO_c_ activity correlates with vasopressor requirement in patients with septic shock. Darcy et al. could show that IDO_c_ activity correlates with severity measures such as the Sequential Organ Failure Assessment (SOFA) score [9]. Zeden and colleagues demonstrated that septic shock coincidence with an exacerbation of kynurenine pathway activity [10]. Additionally, investigations revealed IDO activity to be an independent predictor of disease severity and case fatality in bacteremic patients [11]. Thus, our data add a further translation of previous experimental and clinical work, strengthening the concept that IDO is a novel mediator of arterial vessel relaxation and temporally coupled to the onset and severity of hypotension in human sepsis.

A possible limitation of the current study is that we cannot rule out loss of tryptophan and kynurenine due to deep-freeze storage for several years. However, values at baseline (i.e. before LPS infusion) match those of healthy controls from the literature. Furthermore, as the study is a re-analysis of blood samples from a placebo-controlled interventional trial on pharmacologic p38 MAP kinase inhibition in endotoxemia [4,5], the intake of tryptophan was not standardized. Infusion of LPS in healthy volunteers may be regarded an insufficient model for severe sepsis/ septic shock. This model enabled us nonetheless to study the time course of IDO induction in humans for the first time. Another possible limitation might be the small number of participants. However, due to statistical analysis using advanced random effects linear regression models, in which the respective parameters are “correlated” with each other throughout the whole time course, a small number of study participants should not be crucial. Finally, we cannot exclude an impact of fluid administration (1000 ml saline 0.9% in two patients) on tryptophan and kynurenine levels. While the impact of fluid administration on the concentration of both substances cannot be excluded, calculation of IDO activity should not be affected, as a potential dilution would decrease both amino acids to the same extent.

## Conclusions

In conclusion, our findings support the concept that IDO, the rate-limiting enzyme for tryptophan catabolism, is a novel and previously unrecognized mediator of hypotension that might be particularly significant in human sepsis [12].

## Abbreviations

DBP: Diastolic blood pressure; IDO: Indoleamine 2,3-dioxygenase; LPS: Lipopolysaccharide; MAP: Mean arterial pressure; SBP: Systolic blood pressure; sEsel: Soluble E-selectin; sVCAM-1: Soluble vascular cell adhesion molecule-1.

## Competing interests

The authors declare that they have no competing interests.

## Authors’ contributions

JP, JTK PK analysed the data and wrote the manuscript. CPK contributed to statistical analysis and critically revised the manuscript. JGZ collected the data and critically revised the manuscript; MM supervised the soluble ELISA measurements and critically revised the manuscript. JM and SMB carried out measurements of kynurenine and tryptophan and critically revised the manuscript. All authors read and approved the final manuscript.
